# Evaluation of a Reverse Transcription-Quantitative Polymerase Chain Reaction (RT-qPCR)-Based Microneutralization Assay for Assessing Clinical Human Cytomegalovirus-Neutralizing Antibody Activity

**DOI:** 10.3390/microorganisms12040742

**Published:** 2024-04-06

**Authors:** Jiaao Yu, Maria E. Hasing, Jutta K. Preiksaitis, Xiaoli Pang

**Affiliations:** 1 Department of Laboratory Medicine and Pathology, University of Alberta, Edmonton, AB T6G 1C9, Canada; 2 Department of Medicine, University of Alberta, Edmonton, AB T6G 2G3, Canada; 3 Provincial Laboratory for Public Health, Edmonton, AB T6G 2J2, Canada

**Keywords:** human cytomegalovirus, viral mRNA, neutralization, RT-qPCR, immunostaining assay

## Abstract

Development of a vaccine for human cytomegalovirus (hCMV) is critical because of the severe consequences of infection in congenitally infected newborns and immunocompromised patients. The assessment of hCMV-neutralizing antibody activity is crucial for vaccine development. This study evaluated a RT-qPCR assay targeting the immediate-early gene transcript of hCMV for determining microneutralizing antibody activity. The assay was evaluated for sensitivity, specificity, and precision using endotheliotropic clinical isolate VR1814 that infects fibroblasts, epithelial, and endothelial cells. The RT-qPCR-based neutralization assay was compared with an immunostaining-based neutralization assay using virions present in hCMV-positive urine, saliva, and breast-milk samples. Our results showed that hCMV replication was detectable at 20 h post-infection with a limit of detection of 1 infectious units (IU)/reaction. The RT-qPCR assay had a dynamic range of 1 to 1.0 × 10^4^ IU/reaction, with coefficients of variation ranging from 0.94% to 15.08%. The RT-qPCR results were in high agreement with the immunostaining assay for hCMV-antibody neutralization assessment. Overall, the RT-qPCR neutralization assay is a reliable, rapid, efficient, and sensitive alternative method for evaluating hCMV-neutralizing activity in vitro.

## 1. Introduction

Human cytomegalovirus (hCMV) infection is a common infection. An estimated 50–80% of adults in the United States have been infected with hCMV by the age of 40 [1]. Acute primary infection with hCMV is often asymptomatic followed by lifelong persistence primarily in a latent form in CD34+ hemopoietic progenitor cells and CD14+ monocytes [2]. However, hCMV can cause significant morbidity and even mortality in infants with congenital hCMV infection and immunocompromised hosts [3,4]. In the United States, around 40,000 infants (0.7–1.3% of births) are congenitally infected by hCMV annually, resulting in an estimated 400 death cases and approximately 50% of children with symptoms developing long-term neurological disabilities [3,5]. In immunocompromised hosts infected with HIV, CMV retinitis can lead to blindness [6]. In hematopoietic stem cell and solid organ transplant recipients, CMV infection can result in CMV syndrome, end-organ diseases such as colitis and pneumonitis, and superinfection events [7].

Due to the severe consequences of hCMV infection in these populations, hCMV vaccine development has been recognized as a top priority for public health by the Institute of Medicine in the United States [8]. Several vaccine candidates have been evaluated in clinical trials. A gB/MF59 vaccine candidate developed based on the strain Towne was reported to have an efficacy of 50% against primary hCMV acquisition, which is the highest efficacy of all vaccine candidates in phase II clinical trials [9]. However, a clinical trial of the vaccine in seronegative girls (age 12 to 17 years) demonstrated no statistically significant difference in hCMV infection rates between vaccine and placebo groups [10]. Thus, alternate vaccine candidates are still required.

HCMV can infect a variety of cell types in the human body, typically epithelial cells, endothelial cells, and fibroblasts during primary infection [11]. To enter different cell types, hCMV used different envelope glycoproteins; glycoprotein B (gB) and the trimeric complex gH/gL/gO for entry to fibroblasts and pentameric complex (PC) gH/gL/UL128–131 for epithelial and endothelial cells [12]. Cell-culture-based studies suggest that gB, gH/gL/gO and PC-specific antibodies all had potent neutralizing activities against hCMV in vitro [13,14].However, most of the neutralization studies were based on laboratory-adapted strains such as AD169 or Towne, which are poor representatives of hCMV because the genomes of those strains have mutated extensively, adapting to serial passage in fibroblasts when compared to the genome of low-passage clinical strains [15]. Cui et al. demonstrated that clinical strains of hCMV in primary urine samples were resistant to neutralization by antibodies targeting gB, trimeric complex gH/gL/gO, or PC gH/gL/UL128–131, leading us to hypothesize that the hCMV strains in a wider range of un-passaged original samples, such as urine, saliva, and breast milk may also be neutralization resistant, creating a major barrier for vaccine development [16].

Unlike laboratory-adapted strains that can easily produce high titers of viral particles, hCMV in clinical specimens often have a limited number of viral particles capable of infecting cells in vitro [17]. To investigate the neutralizing antibody activity against clinical strains of hCMV in original clinical samples of different types, a neutralization assay with high sensitivity and specificity is needed. Wang et al. previously described a RT-qPCR-based microneutralization assay as a rapid method to replace traditional staining-based hCMV neutralization assays [18]. The RT-qPCR assay utilizes a pair of primers that span an exon–exon junction of viral RNA, allowing for specific detection of hCMV replication and eliminating interference from neutralized virus. However, there are some challenges in applying this method in clinical studies. Firstly, it is important to note that the RT-qPCR-based neutralization assay was developed using AD169, and therefore the parameters for this assay, such as detection timepoints, may not be directly applicable to clinical hCMV strains. Secondly, for neutralization studies involving clinical hCMV strains, sensitivity is an important parameter to consider as these strains often grow less efficiently in vitro compared to AD169 [17].

Furthermore, it is important to consider the time required to perform neutralization assays in large clinical studies. A fast turnaround time is necessary to accommodate the testing of a large number of samples [19]. Shatzkes et al. proposed using crude cell lysates directly for RT-qPCR neutralization assays without RNA extraction, which further simplifies the procedure [20]. In our study, we optimized and validated a hCMV RT-qPCR-based neutralization assay using crude cell lysates for the first time and determined the sensitivity, specificity, precision, and dynamic range of the assay. The assay was compared in parallel with an immunostaining-based neutralization assay for the measurement of neutralizing antibody activity against both endotheliotropic clinical-isolate strains and hCMV virions present in a variety of clinical-sample types from subjects with active CMV infection.

## 2. Materials and Methods

### 2.1. Cells and Media

MRC-5 (CCL-171) human embryo fibroblasts were obtained from American Type Culture Collection (ATCC; Manassas, VA, USA) and cultivated in Minimum Essential Medium Eagle (MEM; Sigma-Aldrich, Burlington, MA, USA) supplemented with 10% fetal bovine serum (FBS; Gibco, Thermo Fisher Scientific, Waltham, MA, USA), 2 mM L-Glutamine (200 mM, Gibco, Thermo Fisher Scientific, Waltham, MA, USA), and 0.2% Gentamicin (Gibco, Thermo Fisher Scientific, Waltham, MA, USA). ARPE-19 (CRL-2302) human retinal pigment epithelial cells were obtained from ATCC and cultivated in Dulbecco’s Modified Eagle’s Medium (DMEM; Sigma-Aldrich, Burlington, MA, USA) supplemented with 10% FBS, 2.5 mM L-Glutamine. HMEC-1 (CRL-3243) human dermal microvascular endothelium cells were kindly provided by Dr. Allan Murray (University of Alberta, Edmonton, AB, Canada) and cultivated in MCDB 131 (Sigma-Aldrich, Burlington, MA, USA) supplemented with 10% FBS, 10 ng/mL epidermal growth factor (EGF; 10 ng/mL, Sigma-Aldrich, Burlington, MA, USA), 10 nM L-Glutamine and 1 μg/mL Hydrocortisone stock solution (50 μg/mL, Sigma-Aldrich, Burlington, MA, USA).

### 2.2. Viruses and Antibodies

The endotheliotropic HCMV clinical isolate VR1814 (GenBank Sequence Accession: GU179289) was kindly provided by Dr. Elena Percivalle (Fondazione IRCCS Policlinico San Matteo, Pavia, Italy).

Purified normal immunoglobulin G (IgG) Hizentra (Hizentra^®^; CSL Behring, King of Prussia, PA, USA), Cytogam (CytoGam^®^; CSL Behring, King of Prussia, PA, USA), and a panel of human monoclonal antibodies including anti-gB #1 6B4, anti-gB #2 2B11, anti-gH 11B12 and anti-pentamer 8I21 were kindly provided by Pfizer Inc. at Pearl River, NY, USA. Two concentrations of antibodies were used. The low concentrations were selected based on the IC90 values for VR1814 in fibroblasts previously described by Macagno et al. [21]: Cytogam (640 μg/mL for urine or 1280 μg/mL for saliva), 10 μg/mL for anti-gB #1, 7.5 μg/mL for anti-gB#2, 35 μg/mL for anti-gH, and 25 μg/mL for anti-pentamer. The high concentrations of immunoglobulin and antibodies were chosen based on the concentrations used by Cui et al. [16]: Cytogam (1280 μg/mL for urine or 2100 μg/mL for saliva) and 50 μg/mL for anti-gB #1, anti-gB #2, anti-gH, and anti-pentamer.

### 2.3. Clinical Specimens

A total of 34 clinical specimens were obtained from 15 individuals. Urine (n = 9) and saliva (n = 6) were collected from 6 newborn infants who were breastfed by their hCMV-seropositive mothers; urine (n = 1), saliva (n = 2), and breast milk (n = 16) were obtained from 9 hCMV-seropositive breastfeeding mothers. Clinical specimens were collected 1–4 times per infant (median of intervals = 25 days [range = 20–35 days]) and 1–4 times per mother (median of intervals = 32 days [range = 20–97 days]) respectively. The ethics approval for this study was obtained from the University of Alberta Research Ethics Board. [No. Pro00074348].

### 2.4. RT-qPCR Assay

The SYBR Green-based RT-qPCR was previously described by Wang et al. with minor modification [18]. Briefly, primers AGATGTCCTGGCAGAACTCGTC (forward) and TTCTATGCCGCACCATGTCCAC (reverse) were used to target a 62 bp mRNA segment encoded by IE gene IE1 (UL123) across an exon–exon junction. RT-qPCR analysis was carried out by iScript One-Step RT-PCR kits (Bio-Rad, Hercules, CA, USA) according to the manual. The final RT-qPCR mixture contained 10 μL of iTaq universal SYBR^®^ Green reaction mix (2×), 0.25 μL of iScript reverse transcriptase, 300 nM concentrations of each primer, 2 μL of cell lysate, and nuclease-free H_2_O to 20 μL. Thermal cycles were performed on an Applied Biosystems 7500 Fast Real-Time PCR System (ABI, Foster City, CA, USA) under the standard condition as follows: 10 min at 50 °C for reverse-transcription reaction; 1 min at 95 °C for polymerase activation, 40 cycles of amplification (15 s denaturation at 95 °C and 60 s extension at 60 °C), and a default setting of melt-curve analysis at the end. Result reading was conducted during the extension period. Melting curve analysis was performed and melting temperature within the range of 76.8 to 78.8 °C was considered as melting temperature for true positive results.

A standard curve was generated based on the correlation between infectious units of hCMV and Ct values. A 10-fold serial dilution of extracted hCMV RNA from 1 to 10^4^ IU/PCR reaction was analyzed by RT-qPCR to establish dynamic range and a standard curve for quantification.

### 2.5. Immunostaining Assay

Immunostaining was performed as previously described by Abai et al. [22]. Briefly, after hCMV inoculation, cells were fixed with 150 μL of absolute ethanol for 30 min and exposed to 150 μL of PBS for rehydration for 10 min, followed by a 30 min incubation with 150 μL of 5% normal goat serum (Normal goat serum ab7481, Abcam Inc., Waltham, MA, USA) diluted with PBS. 100 μL of 0.1 μg/mL of primary antibody anti-CMV IE1 monoclonal IgG (clone 8B1.2, Millipore Corporation, Oakville, ON, Canada) and 100 μL of a secondary antibody 5/10,000 dilution of biotin-conjugated goat anti-mouse IgG (Invitrogen™, Fisher Scientific, Waltham, MA, USA) with PBS supplemented with 5% casein blocker (Blocker™ casein, ThermoFisher Scientific, Waltham, MA, USA) were successively added to each well and incubated for 90 min respectively. After antibody binding steps, 100 μL of a 2 μg/mL of HRP-Conjugated Streptavidin (Invitrogen™, Fisher Scientific, Waltham, MA, USA) diluted with PBS supplemented with 5% casein blocker was added to each well for 30 min followed by 100 μL of TrueBlue (TrueBlue™ Peroxidase Substrate; Seracare Life Sciences Inc., Milford, MA, USA) for 15 min. Finally, monolayers were rinsed with distilled water followed by absolute ethanol and then dried inside the hood for 10 min. Cells were washed three times with PBS before each step. Stained spots or plaques were scanned with an ImmunoSpot Analyzer from C.T.L (Cellular Technology Limited, Shaker Heights, OH, USA) and counted manually. Each stained spot or plaque was reported as an infectious unit (IU).

### 2.6. Neutralization Assay

On day one, MRC-5 cells (25,000 cells per well), ARPE-19 cells (20,000 cells per well) and HMEC-1 cells (15,000 cells per well) were seeded in 96-well plates. On day two, breast milk and saliva were centrifuged at 20,000× *g* for 5 min and at 10,600× *g* for 2 min respectively to obtain supernatants. Urine samples and supernatants of breast milk and saliva were diluted with 1.5 times of culture medium supplemented with 1% of penicillin-streptomycin and 0.1% of Fungin. 1000 IU of VR1814 or 850 μL of pre-prepared clinical samples were mixed with equal volume of antibodies at indicated concentrations and incubated for 1 h at 37 °C. 200 μL of the antibody-virus mixture was added to each well of 96-well plates followed by centrifugation at 300× *g* for 30 min and then incubated at 37 °C under 2.5% CO_2_ for 20 h for VR1814 or 48 h for clinical specimens unless otherwise indicated. After incubation, hCMV replicates in infected cells were analyzed by RT-qPCR and immunostaining neutralization assay in parallel as described.

### 2.7. Comparison of hCMV RNA Preparation with and without RNA Extraction

A 10-fold serial dilution of VR1814 from 1 to 10^4^ IU/well was used to inoculate MRC-5, ARPE-19, and HMEC-1 cells in 96-well plates. After a 20-h incubation, cells were washed once with 200 μL per well of PBS and either extracted by MagaZorb^®^ Total RNA Mini-Prep Kits (Promega, Madison, WI, USA) with a final volume of 50 μL elute in nuclease-free water or processed by the crude cell lysate as previously described by Shatzkes et al. with some modifications [20]. Briefly, 100 μL of buffer containing 10 mM Tris-HCl, pH 7.4, 0.25% Igepal CA-630 and 150 mM NaCl was added to each well for 10 min at room temperature followed by a vortex on a medium setting (setting 6 out of 8) for 30 s and a 15,000× *g* centrifugation at 20 °C for 2 min to obtain supernatants. hCMV RNA load in the extracted nucleic acid and crude cell lysate supernatant was analyzed by RT-qPCR in triplicate.

### 2.8. Timepoint Optimization for RT-qPCR Analysis

MRC-5, ARPE-19, and HMEC-1 cells were seeded as described under neutralization assay and inoculated by VR1814 (1000 IU/well) or pre-prepared clinical samples. VR1814-infected cells were collected at 0, 1, 3, 5, 8, 10, 13, 16, 18, and 22 h post-infection (h.p.i.) and analyzed by RT-qPCR. The cells infected by clinical samples were collected at 20 and 48 h.p.i. and analyzed by RT-qPCR.

### 2.9. Evaluation of Sensitivity and Precision of the RT-qPCR Assay

The sensitivity was measured eight times by using five concentrations (0.5, 0.4, 0.3, 0.2, 0.1 IU/μL) of extracted VR1814 RNA produced from virus stock. The precision was evaluated in triplicate on three different days using nine hCMV-positive samples with different expected viral levels.

### 2.10. Comparison between the RT-qPCR and Immunostaining Assays for hCMV Infectivity Detection

For sensitivity comparison, three different concentrations (1, 10, and 20 IU/well) of diluted VR1814 were used to inoculate MRC-5 cells in 96-well plates with six replicates for each concentration. Ten replicates of maintenance medium were included as negative controls to determine the specificity. The correlation was determined by a set of diluted VR1814 inoculators (25, 50, 100, 200, 500, 1000, 1500, 2000 IU/well) in MRC-5 cells with each dilution measured in six replicates. After 20 h incubation, the 96-well plates were analyzed separately by the RT-qPCR and immunostaining assays to determine hCMV infectivity. Pearson’s correlation coefficient was calculated to determine the degree of agreement between the two methods, with data from immunostaining plotted on the *y*-axis against data from RT-qPCR on the *x*-axis, and a regression line drawn. The correlation coefficient (*r*) was then calculated using the equation as follow: $r_{xy\;}=\frac{\sum_{i=1}^{n}\;(x_{i}-\bar{x})\;(y_{i}-\bar{y})}{\sqrt{\sum_{i=1}^{n}{\;(x_{i}-\bar{x})}^{2}}\sqrt{\sum_{i=1}^{n}{\;(y_{i}-\bar{y})}^{2}}}$, where *n* is the sample size, $\bar{x},\bar{y}$ are sample means, and $x_{i},y_{i}$ are individual sample points.

The RT-qPCR-based and immunostaining-based neutralization assays were performed in parallel on hCMV-DNA positive clinical samples. A total of 30 clinical samples were included in this comparison. Twenty-one clinical samples were tested in three cell lines, while six samples were tested in two cell lines and three samples were tested in one cell line due to limited volume. Fisher’s exact test was applied to analyze if the performances of two assays were the same using the equation: $p=\frac{\left(a+b\right)!\left(c+d\right)!\left(a+c\right)!\left(b+d\right)!}{a!b!c!d!n!}$, where *a* represents the number of samples detected as positive by both assays, *b* represents the number of samples detected as negative by RT-qPCR but positive by immunostaining, *c* represents the number of samples detected as positive by RT-qPCR but negative by immunostaining, and *d* represents the number of samples detected as negative by both assays. The confidence level is 95% for the Fisher’s exact test so that the definition of statistical significance was *p* < 0.05.

### 2.11. Statistical Analysis

Descriptive statistics and correlation coefficient were obtained from Microsoft Excel (Microsoft Office 365 Proplus, version 1912). Neutralization curves, half maximal inhibitory concentration (IC50), and paired *t* test were plotted and calculated by GraphPad Prism 8 (GraphPad Software, San Diego, CA, USA). Results were considered significant if *p* < 0.05.

## 3. Results

### 3.1. Optimization of the Direct Cell Lysate for RT-qPCR Reaction

First, we tested the cell lysate directly for downstream RT-qPCR as described by Shatzkes et al. [20]. However, negative results were observed from hCMV positive controls. We modified the method by adding a 30 s vortex (a medium setting 6 out of 8) followed by a 2 min centrifugation (15,000× *g*) and only used the lysate supernatant for the PCR reaction. PCR inhibition was remarkably reduced by this modification. The yields and quality of hCMV RNA from the lysate supernatant were compared with a reference hCMV RNA extracted from cell culture using the MagaZorb^®^ Total RNA Mini-Prep Kit to assess its performance in RT-qPCR. There was no statistically significant difference in hCMV RNA yields between two preparations (*p* > 0.05, paired *t* test) when an equivalent of hCMV input ranging from 1 to 10^4^ IU/well was used (Table 1).

**Figure 1:**
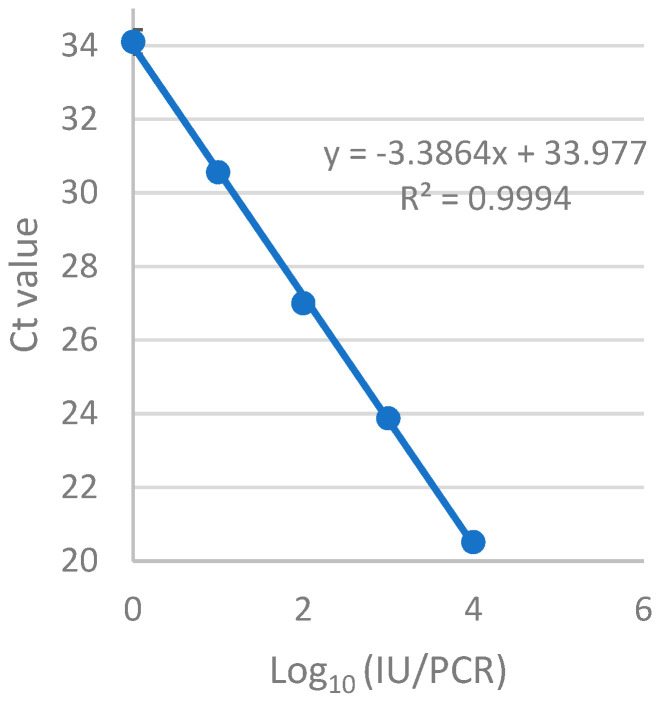
A standard curve of the one-step RT-qPCR assay. The standard RNA from the hCMV immediate-early gene UL123 was extracted from 10-fold serial dilutions of hCMV ranging from 1 to 10^4^ IU/reaction and amplified in the one-step RT-qPCR assay. The quantity of infectious particles was plotted against average cycle numbers.

A good linearity was observed from 1 to 10^4^ IU/reaction with an amplification efficiency of 93.5% for the RT-qPCR assay when the crude cell-lysate supernatant was used to replace RNA extraction (Figure 1). The limit of detection of hCMV by the assay was determined using eight replicates and found to be 1 IU/reaction with 100% sensitivity. As the primers were designed to span an exon–exon junction, cross-reactivity between hCMV RNA and DNA is avoided. Table 2 shows that the coefficient of variation ranged from 15.08% at 5 IU/reaction to 0.94% at 10^4^ IU/reaction, indicating excellent precision.

### 3.2. Optimization of Harvest Timepoint of Cultured hCMV for the RT-qPCR Assay

**Figure 2:**
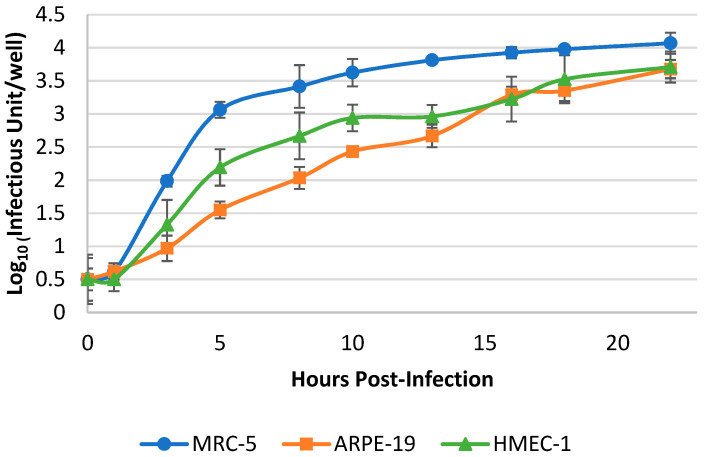
VR1814 proliferation in MRC-5, ARPE-19 and HMEC-1. 1000 IU/well of VR1814 were seeded in MRC-5, ARPE-19, and HMEC-1 in 96-well plates. Culture medium and monolayers were collected at 0, 1, 3, 5, 8, 10, 13, 16, 18, and 22 h.p.i. and analyzed by RT-qPCR with primers targeting the hCMV IE1 gene transcript. Each point represents the mean with the standard deviation of the data set (n = 4).

The kinetic curves of VR1814 in three cell lines were analyzed to determine the earliest time point at which RT-qPCR detection was possible. VR1814 gene transcripts increased faster in fibroblasts than in epithelial and endothelial cells (Figure 2). The RT-qPCR signals of hCMV from the three cell lines showed a 3-log increase at 16 h.p.i. and reached a plateau at 20 h.p.i. This suggests that sufficient hCMV RNA had been produced for RT-qPCR detection at 20 h.p.i.

**Figure 3:**
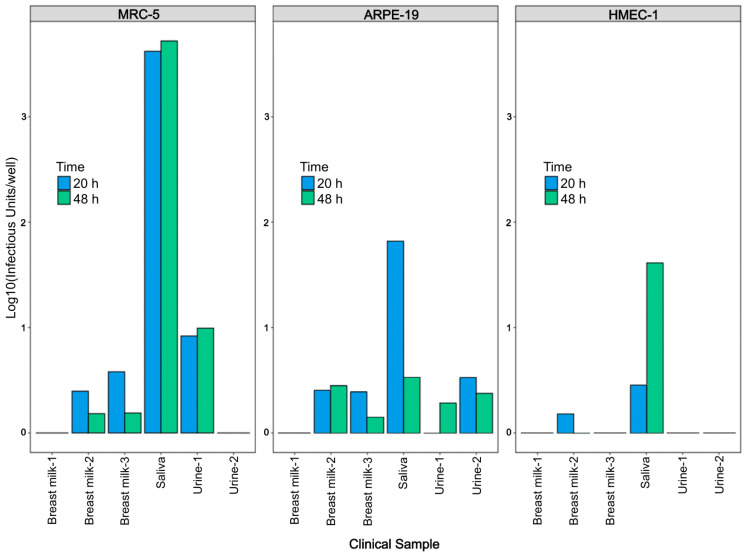
RNA yields of hCMV from clinical samples at 20 vs. 48 h.p.i. by RT-qPCR. 200 μL of hCMV positive samples (3 breast milk, 1 saliva and 2 urine) were used to inoculate MRC-5, ARPE-19, and HMEC-1 in 96-well plates. Culture medium and monolayers were harvested at 20 and 48 h.p.i. and analyzed by RT-qPCR.

Considering the potential differences in cell tropism between VR1814 and clinical strains present in patient samples, we further evaluated the virion proliferation using six different clinical samples (three breast milk, one saliva, and two urine samples) in three cell lines at 20 and 48 h.p.i. The RT-qPCR assay revealed that the overall RNA yields from hCMV in clinical samples at 20 and 48 h.p.i. were lower in cell lines than those of VR1814. The RNA signal from saliva was stronger than that from milk and urine samples across all three cell types at both 20 and 48 h.p.i. However, there was no statistically significant difference between the two time points (*p* > 0.05, paired *t*-test) (Figure 3). These results suggest that 20 h.p.i. is the earliest time point for accurate RT-qPCR assessment of both VR1814 and clinical strains of hCMV in vitro.

### 3.3. Assessment of the Sensitivity, Specificity and Correlation of RT-qPCR and Immunostaining Neutralization Assays

The sensitivity of the RT-qPCR neutralization assay was compared to the immunostaining assay for the detection of hCMV infectivity in MRC-5 cells using VR1814. The RT-qPCR assay had a sensitivity of 1 IU/well, while the immunostaining assay had a sensitivity of 10 IU/well (Table 3). Moreover, the RT-qPCR assay detected hCMV infection in all six replicates at 20, 10, and 1 IU/well, whereas the immunostaining assay detected only two out of six replicates at 10 IU/well with no detection at 1 IU/well inoculation. These results indicate that the RT-qPCR assay is 10 times more sensitive than the immunostaining assay for the assessment of hCMV infectivity.

The RT-qPCR assay demonstrated perfect specificity, with no false positives observed in any of the 10 replicates of negative controls (Table 3). In contrast, the immunostaining assay yielded three false positives out of the 10 replicates of negative controls. These false positives were visually confirmed as stained spots arising from crystalline particle backgrounds. Overall, the RT-qPCR assay showed superior specificity compared to the immunostaining assay.

**Figure 4:**
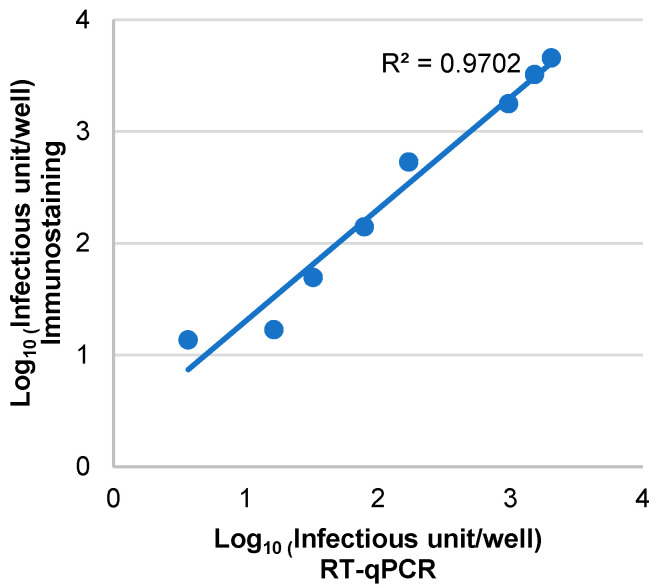
Correlation between the RT-qPCR and immunostaining assays in determining infectious hCMV virions. A series of diluted VR1814 was used to inoculate MRC-5 cells in two 96-well plates. The cells in each plate were analyzed using either RT-qPCR or immunostaining. The results were plotted on a scatter plot, with data from the immunostaining assay on the *y*-axis and data from the RT-qPCR assay on the *x*-axis. A regression line was drawn based on the data (n = 6).

An excellent correlation (R^2^ = 0.97) was observed between the two assays in assessing VR1814 infectivity over a range of viral inputs from 25 to 2000 IU/well in MRC-5 cells (Figure 4). The linear trendline of the plotted points slightly shifted towards the immunostaining side, also indicating that RT-qPCR is more sensitive than immunostaining in quantifying infectious hCMV virions.

### 3.4. Assessment of Antibody Neutralization Activity against VR1814 and hCMV Strains in Primary Clinical Samples by RT-qPCR and Immunostaining Neutralization Assays

**Figure 5:**
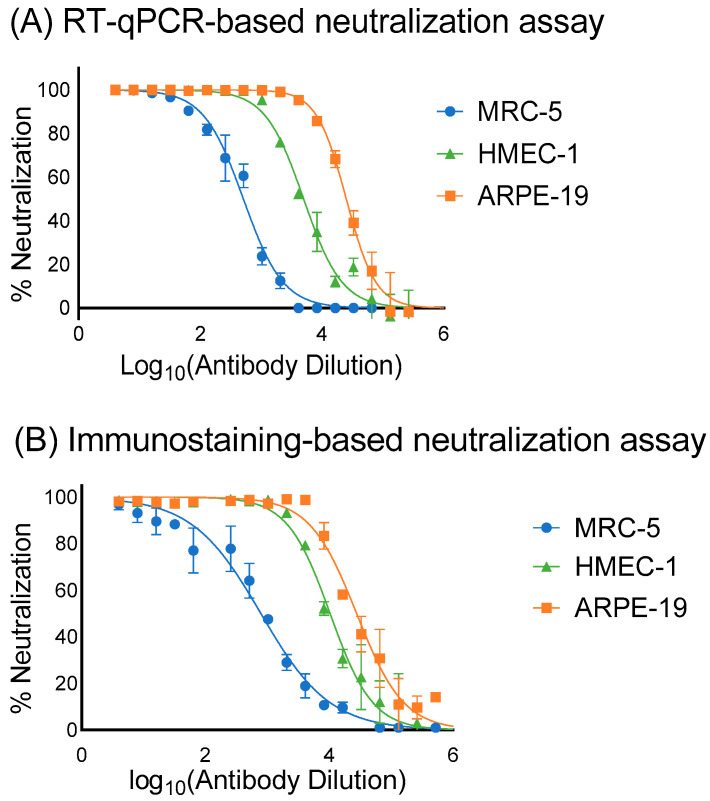
Neutralization efficacy curves of VR1814 generated by (A) RT-qPCR-based and (B) immunostaining-based neutralization assays. A 2-fold serial dilution of IgG Hizentra from 50 mg/mL to 0.38 μg/mL was incubated with an equal volume of 1000 IU of VR1814 for 1 h. The mixture was used to inoculate MRC-5, ARPE-19 and HMEC-1 in 96-well plates and analyzed by (**A**) RT-qPCR and (**B**) immunostaining respectively (n = 3).

The VR1814-neutralizing activity of the commercial human immunoglobulin product Hizentra was evaluated in vitro using both RT-qPCR and immunostaining neutralization assays. The neutralization curves generated from both assays showed good fitness of points in three different cell lines (R^2^ > 0.98) (Figure 5). The IC50 values obtained from the RT-qPCR assay were 393.31 μg/mL in MRC-5 cells and 8.04 μg/mL in ARPE-19 cells, which were comparable to the IC50 values obtained from the immunostaining assay (254.36 and 7.23 μg/mL, respectively) (Table 4). However, a two-fold difference was observed between the two assays in HMEC-1 cells, where the IC50 of the RT-qPCR assay was 40.3 μg/mL and the IC50 of the immunostaining assay was 19.4 μg/mL.

We also compared the neutralization activity of Cytogam and a panel of monoclonal antibodies against clinical strains of hCMV in different primary patient samples using RT-qPCR versus immunostaining neutralization assays. A total of 78 neutralization tests were conducted by the two assays using 30 clinical samples (9 urine and 6 saliva samples from 6 infants and 15 milk samples from breastfeeding mothers) from six mother–infant pairs. Twenty-four (80%) of the 30 clinical samples tested contained infectious hCMV particles (positive in non-antibody controls), and seven out of the 24 (29%) samples could only be cultured in MRC-5 cells. Clinical samples that could successfully infect cell lines often had a higher hCMV DNA level during screening than those that could not infect cell lines, but no clear correlation was observed between viral infectivity and hCMV DNA load in clinical samples.

Of the 78 tests, 59 (75.6%) showed consistent results between the two assays (Table 5), indicating excellent concordance. However, 19 tests were only positive by RT-qPCR but negative by immunostaining. The difference between the results of the two methods was statistically significant (*p* < 0.05, Fisher’s exact test).

To investigate the discordant results, we further examined the accuracy of the RT-qPCR-based neutralization results. Of the 19 discordant results, all could be confirmed as true positives using different cell lines, different sample types from the same patient, transmitter–receiver (mother–infant) relationship, or follow-up sample collections. The infectious viral load in the 19 tests was generally lower than tests with concordant results, which indicates that the discordant results were primarily due to the high sensitivity of the RT-qPCR assay.

**Figure 6:**
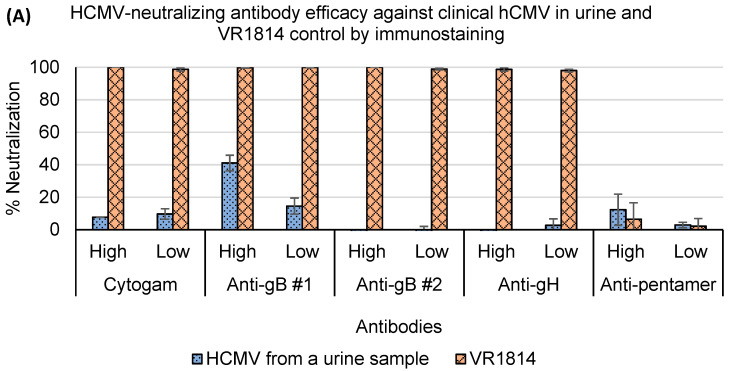

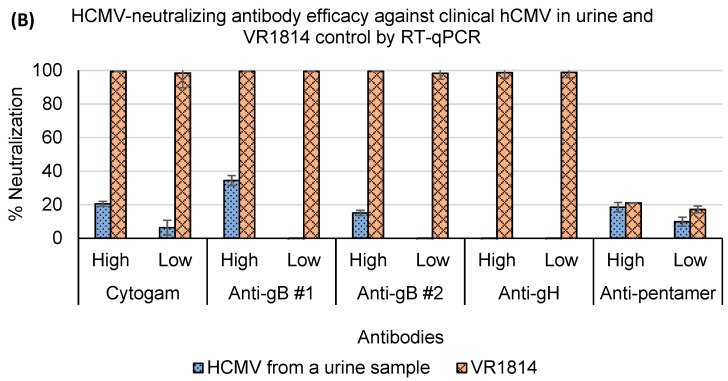
Percentages of antibody neutralization efficacy of hCMV from a representative urine sample and VR1814 detected by (**A**) immunostaining-based and (**B**) RT-qPCR-based neutralization assays. IgG Cytogam at 1280 (high) and 640 μg/mL (low), anti-gB #1 at 50 and 10 μg/mL, anti-gB #2 at 50 and 7.5 μg/mL, anti-gH at 50 and 35 μg/mL, and anti-pentamer at 50 and 25 μg/mL were incubated with equal volume of urine for 1 h and then used to inoculate MRC-5 in 96-well plates. The 96-well plates were assessed by (**A**) RT-qPCR and (**B**) immunostaining respectively after incubation (n = 3).

Thirteen samples from four mother–infant pairs had enough infectious particles to evaluate hCMV neutralization. Detailed neutralization results on these samples which included urine, saliva, and breast-milk samples will be provided in a follow up manuscript. Presented here, is neutralization data from a hCMV-positive urine sample in MRC-5 cells illustrating the high concordance between both assays and the neutralization resistance of virions in uncultured clinical samples (Figure 6). Both assays showed that at least 90% of the positive control VR1814 was neutralized by the antibodies, except for anti-pentamer, which had no neutralizing activity against both clinical strains and VR1814 in MRC-5 cells. However, the Cytogam and the monoclonal antibody panel were found to be ineffective in neutralizing hCMV strains in the urine sample. Only anti-gB #1 showed partial neutralizing activity at a high concentration of 50 μg/mL, with a 41.1% neutralization rate by immunostaining and a 34.4% rate by RT-qPCR (Figure 6).

## 4. Discussion

A neutralization assay is a critical tool for assessing the ability of a vaccine candidate to induce neutralizing antibodies to hCMV that might prevent CMV infection. The traditional “gold standard” for measuring the neutralizing activity of antibodies is the plaque reduction neutralization test (PRNT). In a PRNT, cells that are inoculated by hCMV-antibody mixture have to be cultured for 8 to 12 days to form visible plaques that can be stained and counted under a microscope [23,24]. The dyes that are commonly used to stain cells in the PRNT, such as crystal violet and trypan blue, often fail to reflect the specificity of infected cells, which results in false positive or false negative results [25]. The lack of standardization is also a drawback of traditional PRNT. The number of plaques counted can also be variable among observers, leading to the low agreement in the results obtained by different laboratories [26]. Therefore, although PRNT is still widely used in the study of hCMV, it is time-consuming and has less specificity and sensitivity than alternate methods. The immunostaining-based neutralization assay improves the traditional PRNT. In immunostaining, a dye-conjugated primary antibody is used to specifically bind to the hCMV in cells to increase specificity. Furthermore, an enzyme-conjugated secondary antibody binding to the primary antibody amplifies the signal by catalyzing a chemical reaction that generates a highly visible end product. In this way, the immunostaining-based neutralization assay can visualize a single hCMV-infected cell without the need to form visible plaques. Compared to PRNT, the turnaround time of the immunostaining-based neutralization assay can be shortened to 2 days. The assay has a low coefficient of variation (CV) of around 12% due to its intricate processing steps [22]. The cell tropism of hCMV is another challenge. It is difficult to culture clinical strains of hCMV in certain cell types, such as epithelial cells and endothelial cells, which means the immunostaining signals could be too weak to be captured by plate readers, leading to false negative results [27].

The use of RT-qPCR in neutralization studies offers several advantages, including a short turnaround time, a more standardized procedure, and high sensitivity. The primers of the RT-qPCR used in this study span an exon–exon junction, which ensures our RT-qPCR-based neutralization assay only reflects the replication of hCMV and eliminates the false positive signals generated by neutralized hCMV [18]. A challenge for applying RT-qPCR to a neutralization assay is the tedious RNA-extraction step. A recently published paper described a method for lysis of influenza-infected MDCKs cells, then successfully used the lysate directly for an RT-PCR-based influenza microneutralization assay [20]. However, strong PCR inhibition in the cell lysates was observed when their method was used in our study. Possible reasons for our failure to replicate their results include: (1) the incubation time in our study (20 h) was longer than theirs (6 h). Cells kept proliferating during our extended incubation time and might exceed the maximum number of cells that can effectively be processed by the method described; (2) our study used clinical specimens to inoculate cells rather than the tissue-cultured influenza strain used in their experiments. Many components of clinical samples contain potential inhibitors (e.g., urea in urine or enzyme in saliva) that can impact PCR amplification, that may not be removed by application of cell lysis buffer [28,29]. We optimized a cell-lysate preparation method that dramatically decreased the PCR inhibition initially observed. The RNA yield of the cell lysate generated by this optimized method was comparable to that obtained using a commercial extraction kit. The entire process of cell-lysate generation and running of the RT-qPCR-based hCMV neutralization assay can be completed in 24 h, a significant improvement compared to our reference method, the immunostaining-based neutralization assay.

Wang et al. [18] demonstrated the RT-qPCR assay can be conducted at 6 h.p.i. for laboratory-adapted strains such as AD169. To be applicable for neutralization studies on a wider range of hCMV strains and more cell types, our study found that measurements at 20 h.p.i. are optimal for endotheliotropic clinical isolate VR1814 and hCMV virions when fibroblasts, epithelial, or endothelial cells are used for assessing infectivity of clinical specimens or for microneutralization assays. This is significantly shorter than the 48 h incubation required for the immunostaining assay to generate visible signals.

The optimized RT-qPCR-based assay can generate neutralization curves in MRC-5, ARPE-19, and HMEC-1 cells as reliable as the reference immunostaining-based assay, although a significant discrepancy was observed when calculating IC50 in HMEC-1 cells. IC50 is an important parameter to study drug sensitivity and neutralization resistance, but complex experimental and data-analysis processes make IC50 assay-specific [30,31]. A guideline established by the International Society for Influenza and other Respiratory Virus Diseases indicated that a two- to three-fold difference in IC50 was common for drug-virus inhibition assays [32]. Degraeve et al. reported an interlaboratory variability of IC50 up to 88% [33]. Kalliokoski et al. suggested that using an average of all available IC50 data would be more accurate and applicable than use of a single IC50 value [34]. Therefore, the two-fold difference between RT-qPCR-based and immunostaining-based neutralization assays in IC50 values obtained in HMEC-1 cells would still be considered acceptable.

Clinical samples often contain lower levels of infectious hCMV particles than tissue-culture adapted laboratory hCMV strain. Only about 60% of hCMV-positive samples demonstrated cytopathic effects in cell culture [35]. However, our study found that 80% of screened clinical samples tested positive in cell-culture-based neutralization assays. This high percentage can be attributed to the high sensitivity of RT-qPCR in reflecting viral replication and the signal amplification provided by the secondary antibody in immunostaining. Both methods are more sensitive than the traditional culture method for reading CMV-specific cytopathic effects. For 25% of clinical samples, the hCMV virions were only culturable in MRC-5 cells, highlighting the cell tropism of hCMV and the increased efficiency of viral growth in this cell line. The low viral load in clinical samples and the difficulty in culturing hCMV in ARPE-19 and HMEC-1 cells underscore the importance of using a highly sensitive method for clinical neutralization studies. Our assay, which has demonstrated greater sensitivity than the reference immunostaining assay, is better suited for detecting hCMV infectivity in clinical samples.

The excellent correlation (R^2^ = 0.97) between the RT-qPCR-based and the immunostaining-based neutralization assays illustrated the potential of using our RT-qPCR-based assay to replace the tedious immunostaining-based assay in neutralization studies. A good concordance (75.6%) was obtained between two assays in evaluating the neutralizing activity of antibodies against hCMV in clinical samples. The discrepancy in the comparison was highly likely caused by the high sensitivity of the RT-qPCR assay.

HCMV neutralization resistance was first observed and reported in some mutated strains [36,37]. In 2017, Cui et al. reported that hCMV virions from 10 urine samples were resistant to neutralization with human immunoglobulin and monoclonal antibodies [16]. In the representative urine sample, we described both the RT-qPCR-based and the immunostaining-based neutralization assays supported previous findings that hCMV neutralization resistance is widely present in urine samples and that the anti-gB #1 monoclonal antibody provided the most potent neutralizing activity.

This study has some limitations. The use of the SYBR green system in RT-qPCR reduced running costs, but specificity can also be compromised due to the possibility of SYBR green dye binding to nonspecific DNA fragments that might result in false-positive results. A probe-based RT-qPCR is needed for specificity improvement. Our ability to access clinical samples with hCMV titers high enough to replicate in ARPE-19 and HMEC-1 cells was limited so the comparison between RT-qPCR and immunostaining assays in terms of assessing clinical hCMV neutralizing activity in latter two cell lines requires further study.

## 5. Conclusions

We have optimized an RT-qPCR-based neutralization assay that is reliable for assessing neutralizing activity against both laboratory-adapted hCMV strains and hCMV virions in clinical samples. Our method has the advantage of using RT-qPCR-ready cell lysates, which simplifies the process and has shorter turnaround time compared to immunostaining-based assays. Furthermore, our RT-qPCR-based assay is more sensitive and allows for the detection of low-level hCMV neutralizing activity. Therefore, this assay can be a useful alternative to traditional neutralization assays for studying hCMV neutralization in clinical samples.

## Figures and Tables

**Table 1:** Comparison of hCMV RNA yields obtained by cell-lysate supernatant and a commercial RNA extraction kit from hCMV culture.

hCMV Virions Input (log_10_ IU/Well *)	Mean Values of Crude Cell Lysate Supernatant/RNA Extraction (log_10_ IU/Well)		
	MRC-5	ARPE-19	HMEC-1
4	5.01/5.01	5.17/4.68	5.05/4.91
3	3.58/3.50	4.53/4.32	4.57/4.20
2	2.61/2.36	2.82/2.73	2.90/2.69
1	1.26/1.12	2.17/2.08	2.09/1.73

* Infection unit per well in a log scale.

**Table 2:** Precision of the one-step RT-qPCR assay associated with viral infectious units in cell culture.

IU/Reaction	No. of Samples	SD	%CV
1.1 × 10^4^	9	0.04	0.94
2.6 × 10^3^	9	0.16	4.56
4.3 × 10^2^	9	0.07	2.48
3.0 × 10^2^	9	0.11	4.25
2.9 × 10^2^	9	0.19	7.87
35	9	0.08	5.32
10	9	0.08	7.40
5	9	0.10	13.31
5	9	0.11	15.08

**Table 3:** Assessment of the sensitivity and specificity of hCMV infection by RT-qPCR-based and immunostaining-based neutralization assays.

hCMV Input (IU/Well)	RT-qPCR-Based		Immunostaining-Based	
	Mean ± SD (IU/Well)	No. Pos	Mean ± SD (Viral Particles/Well)	No. Pos
20	22.47 ± 3.58	6/6	4 ± 2.76	6/6
10	10.1 ± 5.99	6/6	0.67 ± 1.03	2/6
1	6.55 ± 5.1	6/6	0	0/6
Neg controls	-	0/10	0.4 ± 0.7	3/10

**Table 4:** Comparison of IC50 values of IgG Hizentra against VR1814 between the RT-qPCR-based and immunostaining-based neutralization assays.

Cell Type	IC50 (μg/mL)	
	RT-qPCR	Immunostaining
MRC-5	393.31	254.36
ARPE-19	8.04	7.23
HMEC-1	40.3	19.4

**Table 5:** The concordance of RT-qPCR-based and immunostaining-based neutralization results on infectious hCMV virion detection in clinical samples.

		RT-qPCR	
		Positive	Negative
Immunostaining	Positive	24	0
	Negative	19	35

## Data Availability

The data presented in this study are available on request from the corresponding author due to confidentiality of sharing clinical information of the patients who were involved in this study. Ethic reviewing and assessment on the individual consents are necessary for protecting the privacy of individual before the data release. However, the original methods and novel development in this study are openly available in MDPI website after the article is published.

## Abbreviations

ATCC	American type culture collection
CV	Coefficient of variation
Ct	cycle threshold
CMV	Cytomegalovirus
DMEM	Dulbecco’s modified eagle’s medium
EGF	Epidermal growth factor
FBS	Fetal bovine serum
gB/H/L/O	Glycoprotein B/H/L/O
IC50	Half maximal inhibitory concentration
h.p.i.	Hours post-infection
hCMV	Human cytomegalovirus
IE	Immediate-early
IgG	Immunoglobulin G
IU	Infectious units
MEM	Minimum essential medium eagle
PBS	Phosphate-buffered saline
PC	Pentameric complex
PCR	Polymerase chain reaction
rcf	relative centrifugal force
RT-qPCR	Reverse transcription-quantitative polymerase chain reaction
SD	Standard deviation
